# iPSYcare: the development of a linked electronic medical records database to study and optimize psychiatric care in Antwerp

**DOI:** 10.1186/s13104-021-05791-6

**Published:** 2021-09-26

**Authors:** Eva Rens, Joris Michielsen, Geert Dom, Roy Remmen, Kris Van den Broeck

**Affiliations:** 1 Collaborative Antwerp Psychiatric Research Institute (CAPRI), University of Antwerp, Antwerp, Belgium; 2 Family Medicine and Population Health (FAMPOP), University of Antwerp, Antwerp, Belgium; 3 Institute of Tropical Medicine (ITM), Antwerp, Belgium

**Keywords:** Psychiatry, Care trajectories, Hospital data, Mental health, Data registry

## Abstract

**Objective:**

The study of care trajectories of psychiatric patients across hospitals was previously not possible in Belgium as each hospital stores its data autonomously, and government-related registrations do not contain a unique identifier or are incomplete. A new longitudinal database called iPSYcare (Improved Psychiatric Care and Research) was therefore constructed in 2021, and links the electronic medical records of patients in psychiatric units of eight hospitals in the Antwerp Province, Belgium. The database provides a wide range of information on patients, care trajectories and delivered care in the region. In a first phase, the database will only contain information about adult patients who were admitted to a hospital or treated by an outreach team and who gave explicit consent. In the future, the database may be expanded to other regions and additional data on outpatient care may be added.

**Results:**

IPSYcare is a close collaboration between the University of Antwerp and hospitals in the province of Antwerp. This paper describes the development of the database, how privacy and ethical issues will be handled, and how the governance of the database will be organized.

## Introduction

Psychiatric case registers systematically and longitudinally collect patient data from mental health services. The richness of the information makes them of interest to researchers and policymakers in mental health care [1]. Digitalization in health care services strongly facilitates the routine collection and storage of patient data, such that the distinction between psychiatric case registers and mental healthcare administrations has largely faded away [2]. Several psychiatric case registers exist today, both on national (e.g., Israel [3], Denmark [4]) and regional level (e.g. London [5], The Middle Netherlands [6]). Some registers contain information on psychiatric inpatients only [3, 4], whereas others cover community mental health services as well [5, 6].

Psychiatric healthcare data are highly relevant for service planning and the evaluation of clinical activities [7]. Linked hospital data can be used to investigate care patterns and patient flows, and to describe the patient population of a region to a granular level. While cohort studies conventionally assess the prevalence of both treated and untreated common mental disorders in the general population, psychiatric healthcare data are an important source of epidemiological information about severe mental disorders with a lower prevalence such as psychotic disorders, as these patients are less likely to participate in survey studies and to acknowledge their symptoms [8]. Moreover, healthcare data may provide additional information on outcomes (e.g., readmissions) and follow-up of care (e.g. [9, 10]).

However, these benefits come with some challenges. Diagnostic information may be less valid compared to research projects in which extensive structured diagnostic instruments are used [11, 12]. Also, according to the current European General Data Protection Regulation (GDPR), patient data may not be used unless patients give explicit informed consent. Finally, a longitudinal and multi-layer database requires extensive expertise in statistics and IT.

There is currently no database in Belgium that includes complete information on service use and psychiatric diagnosis and which allows to reconstruct individual patient care trajectories over facilities. Yet, electronic medical records (EMRs) are nowadays implemented in all hospitals and contain a wealth of information, but are managed autonomously by the hospitals. EMRs contain the national identification number (NIN) as a unique identifier, as well as relevant patient, diagnostic and care information. It is therefore possible to build a new database which combines the EMRs of different hospitals and links patients through their NIN, which can then ultimately serve as a psychiatric case register. A drawback of using EMRs is the lack of uniformity across hospitals. The availability and format of data differ across hospitals, or other definitions or classification systems may be used. Moreover, different software are used to store the EMRs.

This paper describes the development of the iPSYcare (Improved Psychiatric Care and Research) database. The iPSYcare database combines the data from EMRs of five psychiatric hospitals and three psychiatric units in general hospitals in the Antwerp Province (Belgium). The database is governed by the Academic Chair Public Mental Health at the University of Antwerp, which was established in 2019 and funded by the participating hospitals. Over the past 3 years, a consortium was constructed, bringing health care providers and researchers together in order to realize a data-driven mental healthcare. While conducting other relevant research regarding the population’s (un)met mental health needs, this steering committee gave direction to the course of the current project. Special attention is paid to the confidentiality of the data and the privacy of the patients and physicians. The database will be used for scientific research and to optimize mental health care planning in the region.

## Main text

### Coverage and content

All five psychiatric hospitals (Multiversum, OPZ Geel, UPC Duffel, PZ Bethanië, ZNA) and three general hospitals with a psychiatric unit (GZA, AZ Klina, HHZH Lier) in Antwerp Province will be included in the database. Together, these organizations cover about 90% of the psychiatric beds available for this region. Antwerp Province consists of an area of 2876 km^2^ and a population of approximately 1.8 million inhabitants of which 79.9% are adults [13]. One in eight (12.4%) Antwerp people have a non-Belgian nationality and more than one in four (28.6%) is of non-Belgian origin, especially from the Netherlands and Morocco. In the year 2018, a total of 14,592 psychiatric care episodes were recorded in the hospitals in the province of Antwerp [14].

For the inclusion in the iPSYcare database, all adult patients who are admitted to any psychiatric department of the participating hospitals after May 2021 are asked for informed consent for the secondary processing of the data for research purposes. The database includes fulltime inpatients, partial inpatients in day admission as well as outpatients that are treated by a FACT-team (flexible assertive community-based outreach team), but not outpatients who only occasionally come for consultations.

Based on an inventory of the available and relevant data in the EMRs of the participating hospitals, the hospitals and the researchers collectively decided on the content of the new combined database. We aim to record the following data from each adult patient that gave consent:

- hospital and unit information (e.g. hospital identifier, unit specialization, unit capacity)
- medical admission information (e.g. date, referrer, planned vs unplanned admission)
- sociodemographic patient information (e.g. NIN, postal code, year of birth)
- diagnostic information (e.g. main diagnosis, secondary diagnosis, date of diagnoses)
- care information (e.g. prescribed medication, freedom-restricting measures)
- medical discharge information (e.g. date, type of discharge, referral after discharge).

### Working method

The process of setting up the database and reviewing all associated processes was done in close collaboration with the ethical committees, data protection officers and legal advisors from the participating hospitals and the University of Antwerp.

An SQL-database, consisting of different linked tables, is built and stored on a secure server. A technical workgroup was installed, with representatives of the involved healthcare facilities and researchers to finetune the lay-out of the file. Before the first data transfer, the hospitals implement an informed consent procedure in which every patient is informed about the research and is asked for explicit consent for the secondary processing of their medical data. Only data of patients and practitioners who give explicit consent will be included in the database. All the information about the study and participation will also be made available on the websites of the hospitals. Patients are clearly informed about how they can exercise their right of access, rectification, deletion or objection. An opting-out procedure is available for patients who wish to withdraw after giving consent. Patients can then contact the administrative services and data protection officer of the hospital so that they can maintain their anonymity for the researchers.

A first data transfer is planned for the end of 2021. The hospitals will send all the available data of the patients that consented and are treated in any of their wards to the secure SQL-server of the University of Antwerp. Subsequent data transfers take place every 3 months and will only include data of patients that gave consent and were discharged since the previous data transfer.

### Privacy and legal considerations

To ensure that the hospital data can be transferred to the researchers while respecting the privacy of the patients, ‘eHealth’—the Belgian government agency which supports secure information exchange in the healthcare sector—will function as a trusted third party (TTP). More concretely, the process of data transmission includes the following steps:

1. The hospitals retrieve data from their EMR, saving data in a csv-file;
2. The hospitals run a program which encrypts all the information in the csv-file, except the personal data of the patient and the physicians involved (i.e., national identification number of the patients and NIHDI identification number of the healthcare professionals);
3. Using a secure connection, the file is sent to the TTP (i.e., eHealth);
4. The TTP runs an algorithm to encode the personal data. Matching personal data will receive a matching code, so that the data of the same person can be linked across facilities. Irrespective of the source of the data and the time of data delivery, the same algorithm will be used for all data. As medical data are encrypted, the TTP staff is unable to access medical data;
5. Using a secure connection, the TTP sends the files to the server of the University of Antwerp;
6. The researchers are able to decrypt the medical information and the files are loaded into the database. As the personal data are now encoded, the identity of the patient and the physicians involved are unknown for the researchers. However, they are able to link data from one patient that has been admitted in several wards or hospitals by the encoded personal data.

The database is only accessible for the researchers of the Academic Chair Public Mental Health and only via a network connection with the University of Antwerp. The database is locked with a password and any use of the database is logged. All researchers sign a confidentiality contract. The aggregated data will later be made public via an online dashboard, which allows interested parties to run some basic descriptive analyses themselves.

This method is also used by the iCAREdata project (i.e., another research project at the University of Antwerp which uses clinical routine data on out-of-hours care), where it has proven efficacy and ethical soundness [15]. Moreover, the concept and method have been approved by the ethical committees of the University of Antwerp and all involved hospitals (nr. B3002020000157) and by the Belgian National Information Security Committee, guaranteeing a safe method that respects both ethical and juridical regulations. The recommendations of a “small cell risk analysis” will be followed to increase safety of the data and secure patient’s privacy.

### Governance

The database is governed by a steering committee, consisting of the researchers and representatives of the participating hospitals. A collaboration agreement was developed in close collaboration with the legal office and the data protection officer (DPO) of the University of Antwerp, and the DPO’s, ethical representatives and legal experts of the involved hospitals, including agreements on the usage rights on the data and future analyses, the output, and liability and dispute resolution. The steering committee ranks and prioritizes the research questions that will be investigated using the database. For each research question, the researchers will develop a protocol summarizing the variables of interest and the analyses that will be conducted, that will be submitted to the different ethical committees. Hospital representatives have the right to decide whether or not the researchers may use the data from their hospital to investigate the research question. The output of the analyses will be first presented to the members of the steering committee. Efforts will be made to actively involve patients, for example through patient advocates in the steering committee.

## Limitations

A drawback is that the database is limited to inpatient care and does not provide information on a national level. However, the possibility of including outpatient care and psychiatric care in other regions will be considered in a later phase. In the future, record linkage with other registers (e.g., IMA) would be possible as well. It is a technical challenge that the availability and format of the data in the EMRs are not the same across hospitals, but the data will be standardized if possible. A possible bottleneck is the requirement for explicit patient consent. Given the target group, it is likely that a significant proportion of patients will not give consent or will not be able to give informed consent at the time of admission. Nevertheless, at the request of the National Security Committee and strictly following the GDPR, the decision was made to start the research project with an opt-in procedure. If it turns out later that the number of refusals is high, a new ethical application will be submitted on the grounds that explicit consent poses a threat to the validity and reliability of the research. In that case, an opt-out procedure will be requested: all patients are still actively informed but are automatically included unless they actively invoke their right of objection or deletion.

## Data Availability

There is currently no data to be shared yet. The raw data of the database will only be accessible to the researchers. At a later stage, an online open-access dashboard of the aggregated data will be considered.

## Abbreviations

IPSYcare: Improved Psychiatric Care and Research
GDPR: European General Data Protection Regulation
MPD: Minimal Psychiatric Data
IMA: Intermutualistic Agency
EMRs: Electronic medical records
NIN: National Identification Number (or National Insurance Number)
TTP: Trusted Third Party
